# β-Galactosylceramidase Deficiency Causes Upregulation of Long Pentraxin-3 in the Central Nervous System of Krabbe Patients and *Twitcher* Mice

**DOI:** 10.3390/ijms23169436

**Published:** 2022-08-21

**Authors:** Daniela Coltrini, Adwaid Manu Krishna Chandran, Mirella Belleri, Pietro L. Poliani, Manuela Cominelli, Francesca Pagani, Miriam Capra, Stefano Calza, Simona Prioni, Laura Mauri, Alessandro Prinetti, Julia K. Kofler, Maria L. Escolar, Marco Presta

**Affiliations:** 1 Department of Molecular and Translational Medicine, School of Medicine, University of Brescia, 25123 Brescia, Italymarco.prestanibs.it (M.P.); 2 Department of Medical Biotechnology and Translational Medicine, University of Milan, 20133 Milan, Italy; 3 Department of Pediatrics, University of Pittsburgh School of Medicine, Pittsburgh, PA 15224-1334, USA

**Keywords:** CNS, GALC, Krabbe disease, neuroinflammation, PTX3, sphingolipidosis

## Abstract

Globoid cell leukodystrophy (GLD), or Krabbe disease, is a neurodegenerative sphingolipidosis caused by genetic deficiency of lysosomal *β-galactosylceramidase* (*GALC*), characterized by neuroinflammation and demyelination of the central (CNS) and peripheral nervous system. The acute phase protein long pentraxin-3 (PTX3) is a soluble pattern recognition receptor and a regulator of innate immunity. Growing evidence points to the involvement of PTX3 in neurodegeneration. However, the expression and role of PTX3 in the neurodegenerative/neuroinflammatory processes that characterize GLD remain unexplored. Here, immunohistochemical analysis of brain samples from Krabbe patients showed that macrophages and globoid cells are intensely immunoreactive for PTX3. Accordingly, *Ptx3* expression increases throughout the course of the disease in the cerebrum, cerebellum, and spinal cord of GALC-deficient *twitcher* (*Galc^twi/twi^*) mice, an authentic animal model of GLD. This was paralleled by the upregulation of proinflammatory genes and M1-polarized macrophage/microglia markers and of the levels of PTX3 protein in CNS and plasma of *twitcher* animals. Crossing of *Galc^twi/twi^* mice with transgenic *PTX3* overexpressing animals (*hPTX3* mice) demonstrated that constitutive PTX3 overexpression reduced the severity of clinical signs and the upregulation of proinflammatory genes in the spinal cord of P35 *hPTX3*/*Galc^twi/twi^* mice when compared to *Galc^twi/twi^* littermates, leading to a limited increase of their life span. However, this occurred in the absence of a significant impact on the histopathological findings and on the accumulation of the neurotoxic metabolite psychosine when evaluated at this late time point of the disease. In conclusion, our results provide the first evidence that PTX3 is produced in the CNS of GALC-deficient Krabbe patients and *twitcher* mice. PTX3 may exert a protective role by reducing the neuroinflammatory response that occurs in the spinal cord of GALC-deficient animals.

## 1. Introduction

Globoid cell leukodystrophy (GLD), or Krabbe disease, is an autosomal recessive neurodegenerative sphingolipidosis caused by genetic deficiency of the lysosomal hydrolase *β-galactosylceramidase* (*GALC*) [1]. GALC degrades galactosylceramide (a major component of myelin) and other terminal β-galactose-containing sphingolipids, including β-galactosylsphingosine (psychosine). The pathogenesis of GLD has been proposed to arise from the accumulation of the neurotoxic metabolite psychosine present at high levels in the CNS of Krabbe patients [2,3,4,5]. The disease is characterized by neuroinflammation and loss of oligodendroglia and Schwann cells, leading to demyelination of the brain, spinal cord, nerve roots and peripheral nerves [6]. A pathognomonic feature of GLD is the presence in the white matter of globoid cells [7,8], giant multinucleated cells likely originated from resident microglia [9]. Clinically, GLD may manifest in early infancy with fatal neurological dysfunctions [6,10,11]. The standard of care for this disease is hematopoietic stem cell transplantation [12,13].

The acute phase protein long pentraxin-3 (PTX3) is an important regulator of peripheral innate immunity and a key mediator of inflammation during cardiovascular and cerebrovascular diseases [14]. Growing evidence points to the involvement of PTX3 in neurodegenerative disorders [15]. *PTX3* expression is upregulated in the central nervous system (CNS) following pro-inflammatory cytokine stimulation [16], seizure-induced neurodegeneration [17], and ischemia [18]. Accordingly, peripheral PTX3 levels are increased after experimental stroke in mice [19], and plasma PTX3 levels correlate with mortality after ischemic stroke in humans [20]. In addition, serum levels of PTX3 have been proposed as a novel biochemical marker in Parkinson’s disease [21]. Despite this evidence, no data are available about the expression and role of PTX3 in sphingolipid disorders, including GLD.

Here, PTX3 production was investigated in the brain of Krabbe patients and in the CNS of GALC-deficient *twitcher* (*Galc^twi/twi^*) mice, an authentic murine model of GLD [22,23]. Analysis of brain samples from Krabbe patients demonstrated that macrophages and globoid cells are intensely immunoreactive for PTX3. Accordingly, *twitcher* mice are characterized by the progressive upregulation of *Ptx3* expression along the CNS caudal-rostral axis. This was paralleled by the upregulation of various proinflammatory genes and M1-polarized macrophage/microglia markers, with no changes in the peripheral organs. Crossing of *Galc^twi/twi^* mice with transgenic *PTX3* overexpressing animals (*hPTX3* mice) caused an amelioration of clinical features and attenuated spinal cord inflammation in *hPTX3*/*Galc^twi/twi^* offspring when compared to *Galc^twi/twi^* littermates at postnatal day P35, when animals show clear neurologic defects. However, *PTX3* overexpression did ameliorate the histopathological findings and did not affect psychosine accumulation in the CNS of GALC-deficient animals when evaluated at this late stage of the disease. 

Our results demonstrate that PTX3 is produced in the CNS of GALC-deficient Krabbe patients and *twitcher* mice and point to a possible protective role of this immune modulator by reducing, at least in part, the neuroinflammatory response that characterizes GLD.

## 2. Results

### 2.1. PTX3 Immunoreactivity in the Brain of Krabbe Patients

**Figure 1:**
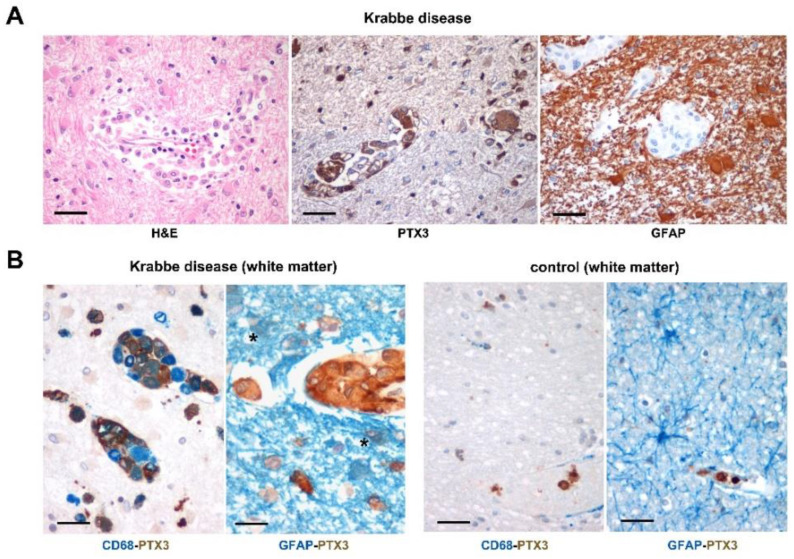
PTX3 immunolocalization in the brain of Krabbe patients. (**A**) Representative images from Krabbe brain cortex specimens showing the classical histopathological hallmarks of the disease in the white matter, with diffuse infiltration of macrophages (globoid cells) mainly distributed around the blood vessels (left image, H&E). Immunostains for PTX3 (brown) and GFAP (brown, middle and right images, respectively) show intense and diffuse immunoreactivity of PTX3 and gliosis. (**B**) Double immunostainings from Krabbe and control brain cortex samples. The large majority of CD68^+^ monocyte-derived cells (blue) are intensely immunoreactive for PTX3 (brown) within the white matter of Krabbe patients (left image), while reactive GFAP^+^ astrocytes (blue) expressing PTX3 (brown) are barely detected (middle left image; asterisk). Conversely, no PTX3^+^ or CD68^+^ cells were detected in these samples, except for rare circulating CD68^-^PTX3^+^ monocytes within the vessels (middle right image), and PTX3 was not detected in resident GFAP^+^ astrocytes from control matched brains (right image). Original magnification: 40× (**A**); 60× (**B**). Scale bars: 50 μm (**A**); 30 μm (**B**).

To assess whether GALC deficiency may cause PTX3 upregulation in human CNS, we analyzed autopsy brain cortex samples from nine Krabbe patients (Table S1) and two matched controls. Most of the Krabbe samples showed the classical histopathological hallmarks of the disease, such as diffuse demyelination along with marked loss of oligodendrocytes, intense reactive gliosis evidenced by the diffuse immunoreactivity of the astrocytic marker GFAP, and infiltration of globoid cells (Figure 1A). Previous observations had shown that activated astrocytes and microglia represent a major source of PTX3 in the brain [16,24]. Accordingly, single immunostains indicated that GLD macrophages and globoid cells are diffusely and intensely immunoreactive for PTX3. Double immunostains confirmed that PTX3 is mainly expressed within macrophages and globoid cells in Krabbe samples, its positivity being related to the extent of pathology (i.e., presence of perivascular globoid cells) and of GFAP-positive astrocytic gliosis (Table 1), while GFAP/PTX3 double positive astrocytes were barely detected. Control brain samples did not show double positive PTX3 immunoreactive cells (Figure 1B). Overall, these findings demonstrate that PTX3 is upregulated in the brain of GLD patients, its immunoreactivity being mainly expressed in monocyte-derived cells and related to the extent of the pathology.

### 2.2. Ptx3 Upregulation in Twitcher CNS

**Figure 2:**
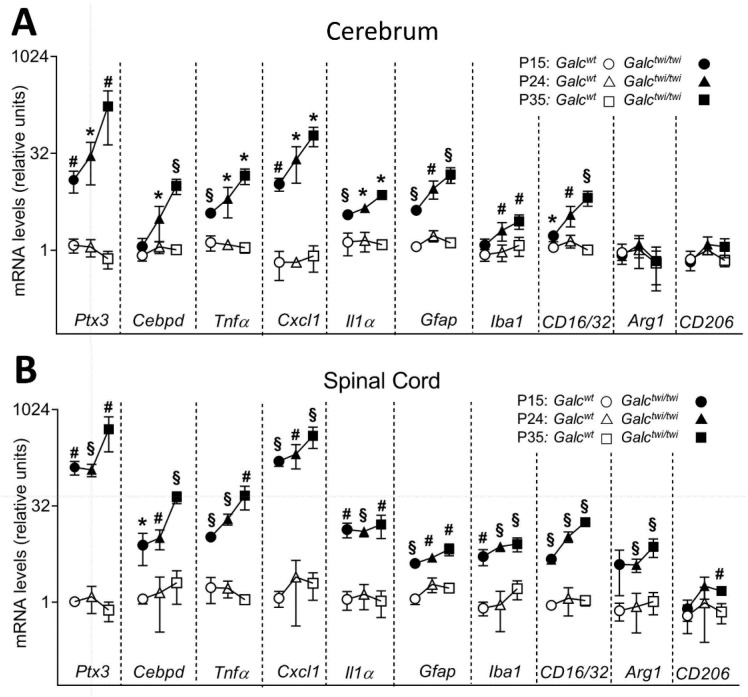
*Ptx3* and proinflammatory gene expression in the cerebrum and spinal cord of *twitcher* mice. Steady state mRNA levels of the indicated genes were evaluated by qPCR in the cerebrum (**A**) and spinal cord (**B**) of *Galc^wt^* and *Galc^twi/twi^* mice harvested at P15, P24 and P35. Data were normalized to *Gapdh* expression and are the mean ± S.D. of 3–6 animals per group, * *p* < 0.05; # *p* < 0.01; § *p* < 0.001, *Galc^wt^ versus Galc^twi/twi^* mice, Student’s *t*-test.

Based on the data obtained in Krabbe patients, the expression of *Ptx3* was investigated in the CNS of GALC-deficient *twitcher* (*Galc^twi/twi^*) mice, an authentic murine model of GLD [22,23]. In a first set of experiments, *Ptx3* expression was assessed by qPCR analysis in the cerebrum of littermate *Galc^wt^* and homozygous *Galc^twi/twi^* mice. Measurements were performed at postnatal day P15 before the onset of evident neurologic signs, at P24 when pathologic alterations were occasionally detectable, and at P35 when *Galc^twi/twi^* mice showed clear neurologic defects, including tremor and hind limb paralysis [23,25,26]. As shown in Figure 2A, the brain levels of *Ptx3* transcripts were already increased at P15, to further increase at P24 and P35. This was paralleled by the upregulation of the inflammatory cytokines *Tnfα*, *Cxcl1* and *Il1*α and of the CCAAT/enhancer binding protein delta (*Cebpd*) gene, which encodes for a transcription factor mediating *PTX3* expression in astrocytes during neuroinflammation [27]. Accordingly, a progressive increase in the levels of the astrocytic marker of gliosis *Gfap* and the microglial marker *ionized calcium-binding adaptor molecule-1* (*Iba1*) [28] occurred in *Galc^twi/twi^* cerebrum when compared to controls. 

In keeping with the neuroinflammatory response that occurs in *twitcher* brain, we observed an increase in the levels of expression of the classical M1 macrophage polarization/microglia markers *Fc receptor, IgG, low affinity III* and *II (Fcgr3/CD16* and *Fcgr2/CD32)* [29] with no changes in the expression of the alternative M2 macrophage polarization markers *arginase-1* (*Arg1*) and *mannose receptor C-type1* (*Mrc1*/*CD206*) [30]. A similar transcriptional profile was observed in the cerebellum (Figure S1) and spinal cord (Figure 2B) of *Galc^twi/twi^* mice that also showed an increase in the M2 polarization markers *Arg1* and *CD206*. Notably, *Ptx3* mRNA levels in the spinal cord of *Galc^twi/twi^* mice were significantly higher than those measured in the cerebrum and cerebellum counterparts at all time points investigated.

**Figure 3:**
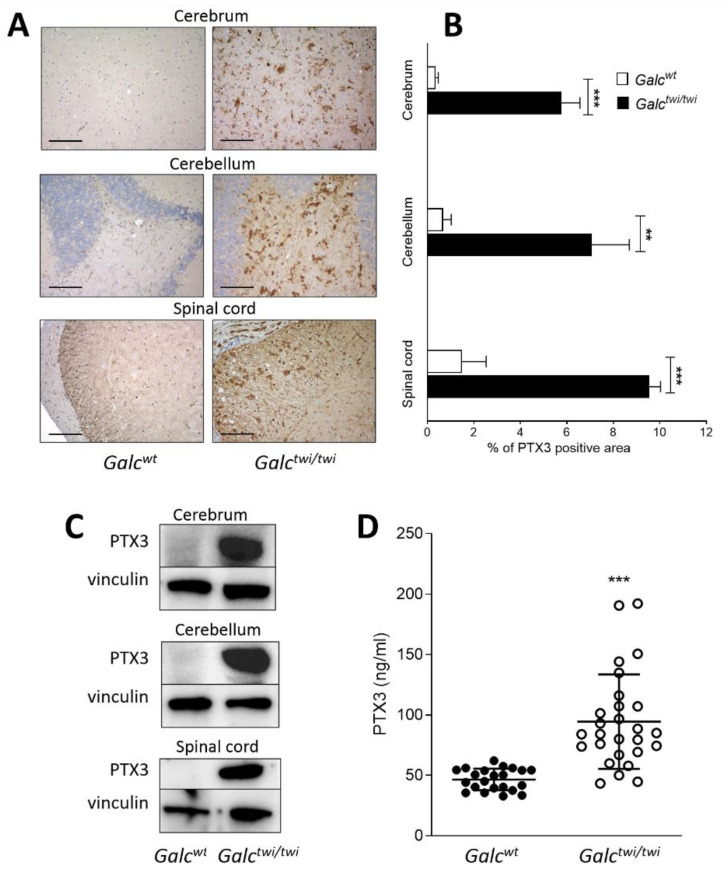
PTX3 protein levels in *twitcher* mice. (**A**) Immunohistochemical analysis shows an increased PTX3 immunoreactivity (brown) in the CNS of P35 *Galc^twi/twi^* mice when compared to control littermates; 20× magnification; scale bar: 100 μm. (**B**) Image analysis of PTX3 positive area. Data are the mean ± S.D. of 3 samples per experimental group (5 fields/sample). (**C**) Western blot analysis of PTX3 immunoreactivity in the CNS of *Galc^wt^* and *Galc^twi/twi^* mice at P35. Vinculin was used as a loading control. (**D**) Blood samples were collected from *Galc^wt^* (n = 22) and *Galc^twi/twi^* mice (n = 27) at P28–34. Then, serum levels of PTX3 were assessed by ELISA. Each point represents one animal. Data are shown as mean ± S.D. ** *p* < 0.01; *** *p* < 0.001, Student’s *t* test.

*Ptx3* upregulation in the CNS of *twitcher* mice resulted in high levels of PTX3 protein in the cerebrum, cerebellum, and spinal cord of P35 *twitcher* mice when compared to control littermates as assessed by immunohistochemistry and Western blot analysis (Figure 3A–C). In addition, the levels of PTX3 in the blood of P35 *twitcher* mice were significantly increased when compared to *Galc^wt^* animals (Figure 3D).

**Figure 4:**
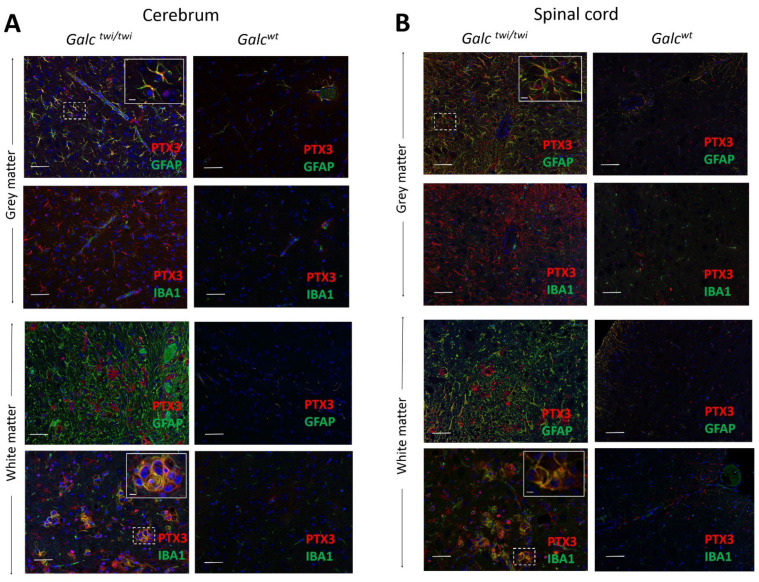
PTX3 immunolocalization in the CNS of *twitcher* mice. Paraffin-embedded sections of the white and gray matter of the brain cortex (**A**) and spinal cord (**B**) of P35 *Galc^twi/twi^* mice were double-immunostained with anti-PTX3/GFAP or anti-PTX3/IBA1 antibodies. Inserts show enlarged areas marked by dashed squares. Scale bar, 50 μm; scale bar in inserts, 5 μm.

On this basis, immunohistochemical analysis was performed to identify PTX3-positive cells in the CNS of *twitcher* animals. As shown in Figure 4, double immunostains indicated that PTX3 immunoreactivity was prevalently detectable in IBA1-positive globoid cells in the white matter of the brain cortex and spinal cord of *Galc^twi/twi^* animals, no significant immunoreactivity being observed in control samples. In addition, PTX3 immunoreactivity was also present in GFAP-positive astrocytes of the gray matter of the CNS of *twitcher* mice, but not of *Galc^wt^* animals that lacked signs of gliosis (Figure 4). Similar results were obtained in *twitcher* cerebellum samples (Figure S2). 

At variance with the results obtained by the analysis of the *twitcher* CNS, no *Ptx3* upregulation occur in the peripheral tissues of *Galc^twi/twi^* mice, including kidney, liver, and lungs, in which only limited psychosine accumulation and inflammatory responses occur because of GALC deficiency [23,25,31,32,33] (Figure S3). Nevertheless, in keeping with the observation that the serum levels of PTX3 are increased after stroke [19,20] and in Parkinson’s disease [21], the levels of PTX3 in the blood of P35 *twitcher* mice were significantly increased when compared to *Galc^wt^* animals (Figure 3D).

### 2.3. PTX3 Overexpression Reduces Clinical Symptoms and Spinal Cord Inflammation in Twitcher Mice

TgN(Tie2-hPTX3) mice (*hPTX3* mice) ubiquitously express human *PTX3* under the control of the *Tie2* promoter, leading to a significant accumulation of the PTX3 protein in serum and tissues that occurs from birth throughout the whole life of the transgenic mice [34]. The constitutive *hPTX3* expression does not result in apparent defects in embryonic development; adult animals are normal and fertile, and no macroscopic or microscopic morphological abnormalities were observed in organs and tissues of *hPTX3* mice [34].

On this basis, to assess a possible impact of the constitutive PTX3 overexpression in *twitcher* mice, syngeneic *PTX3*-overexpressing *hPTX3* male mice were crossed with female *Galc^twi/+^* animals to obtain *hPTX3/Galc^twi/+^* breeders that were then crossed to generate *hPTX3/Galc^wt^* and *hPTX3/Galc^twi/twi^* animals. Next, *hPTX3/Galc^wt^* and *hPTX3/Galc^twi/twi^* mice were compared to the corresponding *Galc^wt^* and *Galc^twi/twi^* littermates. qPCR analysis using oligonucleotide primers designed to recognize simultaneously both human and murine PTX3 transcripts (Table S2) indicated that the levels of *hPTX3* mRNA in the cerebrum of *hPTX3/Galc^wt^* mice were like the levels of the murine *Ptx3* transcripts measured in *Galc^twi/twi^* mice at P35. In addition, *hPTX3* overexpression together with the upregulation of its murine counterpart resulted in a cumulative increase in their mRNA levels in the brain of P35 *hPTX3/Galc^twi/twi^* mice when compared to *Galc^twi/twi^* littermates (Figure S4A). 

**Figure 5:**
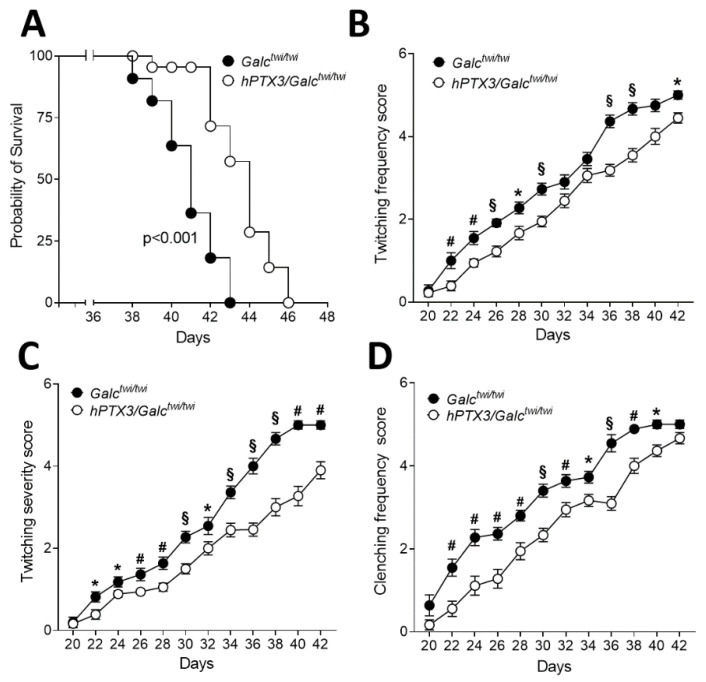
Impact of *hPTX3* overexpression on the clinical features of *twitcher* mice. (**A**) The life span of *hPTX3/Galc^twi/twi^* mice (n = 15) was compared to that of *Galc^twi/twi^* mice (n = 11). *hPTX3* overexpressing animals had a significant extension of life span compared to control *twitcher* mice. *p* < 0.001, Gehan–Breslow–Wilcoxon test. (**B**–**D**) The extent of frequency (**B**) and severity (**C**) of twitching and hind limbs clenching frequency (**D**) were scored in *hPTX3/Galc^twi/twi^* mice (n = 15) and *Galc^twi/twi^* mice (n = 11). No neurological signs were observed before day P20 in both groups of animals. No animal death and clinical symptoms were observed in *hPTX3/Galc^wt^* and *Galc^wt^* mice throughout the whole experimental period. * *p* < 0.05; # *p* < 0.01; § *p* < 0.001, Student’s t-test.

As shown in Figure S4C,D, constitutive *hPTX3* overexpression did not affect the body weight gain of both *hPTX3/Galc^wt^* and *hPTX3/Galc^twi/twi^* mice when compared to the corresponding control *Galc^wt^* and *Galc^twi/twi^* animals, with *Galc* deficiency leading to a similar decrease in body weight starting from approximately day P20 and P28 both in the absence or in the presence of constitutive *hPTX3* overexpression in male and female animals, respectively. Nevertheless, *hPTX3* overexpression resulted in statistically significant, albeit limited 4-day increases in the overall survival of *twitcher* animals, extending their life span from 42 to 46 days (*p* < 0.001) (Figure 5A), thus suggesting that PTX3 may exert an impact on the disease course. Indeed, when compared to *Galc^twi/twi^* animals, *hPTX3/Galc^twi/twi^* mice showed a reduced frequency and severity of twitching, which appeared around day P22 in both groups of animals (Figure 5B,C). In addition, *hPTX3/Galc^twi/twi^* mice displayed a less severe atypical tail suspension reflex (hind limbs clenching) (Figure 5D). No animal death and appearance of clinical symptoms were instead observed in *hPTX3/Galc^wt^* and *Galc^wt^* mice during the same investigation period.

**Figure 6:**
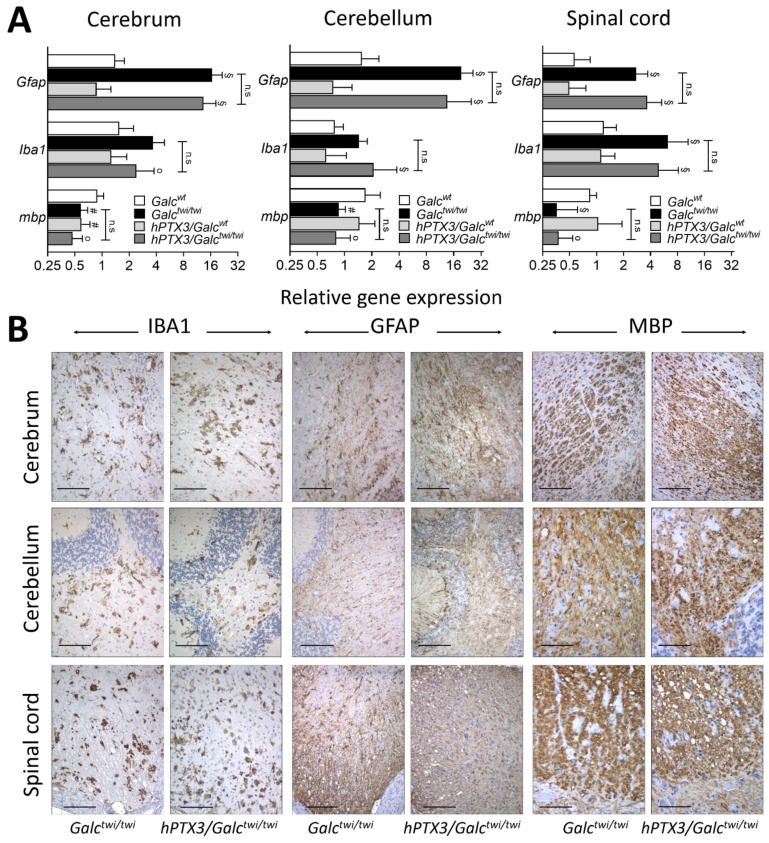
Impact of *hPTX3* overexpression on histopathological features of *twitcher* mice. (**A**) mRNA levels of the indicated genes were evaluated by qPCR in the CNS of P35 *hPTX3/Galc^wt^* and *hPTX3/Galc^twi/twi^* mice and compared to the corresponding control *Galc^wt^* and *Galc^twi/twi^* animals. Data were normalized to *Gapdh* expression and are the mean ± SEM of 7–10 animals per group. n.s., not significant for *Galc^twi/twi^ versus hPTX3/Galc^twi/twi^* mice; ° *p* < 0.05, # *p* < 0.01, § *p* < 0.001 for *Galc^wt^* *versus Galc^twi/twi^* mice or for *hPTX3/Galc^wt^ versus hPTX3/Galc^twi/twi^* mice. One-way ANOVA with post hoc comparisons with adjustment for multiple comparisons (Sidak). (**B**) Immunohistochemical analysis shows similar IBA1, GFAP and MBP immunoreactivity (brown) in the CNS of P35 *hPTX3/Galc^twi/twi^* mice when compared to *Galc^twi/twi^* animals; 20× magnification; scale bar: 100 μm.

On this basis, we assessed the levels of expression of the microglial marker *Iba1*, of the astrocytic marker of gliosis *Gfap*, and of the myelin basic protein-encoding gene *mbp* in the cerebrum, cerebellum, and spinal cord of the different experimental groups at P35. As shown in Figure 6A, GALC deficiency caused a similar upregulation of *Iba1* and *Gfap* genes and downregulation of *mbp* in the CNS of *hPTX3/Galc^twi/twi^* and *Galc^twi/twi^* mice at this late stage of the disease when compared to *Galc^wt^* and *hPTX3/Galc^wt^* animals. Accordingly, immunohistochemical analysis demonstrated that the IBA1-positive microglia infiltrate, GFAP-positive gliosis, and myelin degradation of MBP-positive fibers were present to a similar extent in the CNS of P35 *hPTX3/Galc^twi/twi^* and *Galc^twi/twi^* mice (Figure 6B).

In keeping with the lack of a significant impact of constitutive *hPTX3* overexpression on the histopathological features of the CNS of GALC-deficient animals, the levels of the neurotoxic GALC substrate psychosine were increased to a similar extent in the CNS of *Galc^twi/twi^* and *hPTX3/Galc^twi/twi^* animals (Table 2).

**Figure 7:**
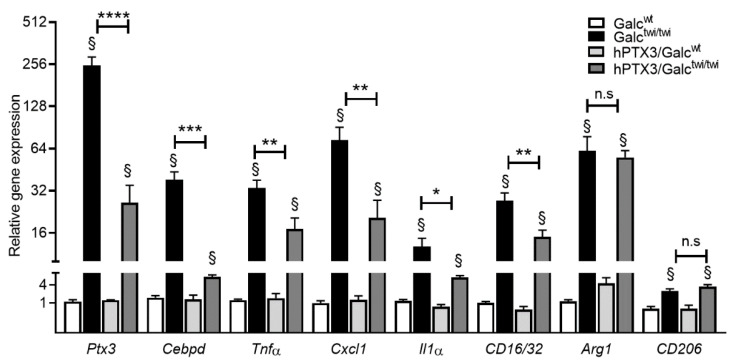
qPCR analysis of the spinal cord of *hPTX3/Galc^wt^* and *hPTX3/Galc^twi/twi^* mice. Steady state mRNA levels of the indicated genes were evaluated by qPCR in the spinal cord of *hPTX3/Galc^wt^* and *hPTX3/Galc^twi/twi^* mice and compared to those measured in the corresponding control *Galc^wt^* and *Galc^twi/twi^* mice harvested at P35. Data were normalized to *Gapdh* expression and are the mean ± SEM of 7–10 animals per group. n.s., not significant; * *p* < 0.1; ** *p* < 0.05; *** *p* < 0.01, **** *p* < 0.001 for *Galc^twi/twi^ versus hPTX3/Galc^twi/twi^* mice; §, *p* < 0.001 for *Galc^wt^* *versus Galc^twi/twi^* mice or for hPTX3/*Galc^wt^ versus hPTX3/Galc^twi/twi^* animals. One-way ANOVA with post hoc comparisons with adjustment for multiple comparisons (Sidak).

Finally, qPCR analysis of cerebrum, cerebellum, and spinal cord of P35 animals was performed to assess the impact of constitutive *hPTX3* overexpression on the inflammatory response of the CNS in GALC-deficient mice. As shown in Figure 7, the expression of the inflammatory mediators *Tnfα*, *Il1α*, and *Cxcl1* and of the M1 polarization macrophage/microglia markers *CD16/CD32*, albeit still higher than that observed in control wild type animals, was significantly reduced in the spinal cord of *hPTX3/Galc^twi/twi^* mice when compared to the *Galc^twi/twi^* counterpart, whereas *Arg1* and *CD206* expression remained unchanged. 

At variance, no differences in gene expression were detected in the cerebrum and cerebellum of the two groups of animals, the only exceptions being represented by a slight increase in the expression of *TNFα* in the cerebrum of *hPTX3/Galc^twi/twi^* mice when compared to *Galc^twi/twi^* animals and a decreased cerebellar expression of the M2 polarization markers *Arg1* and *CD206* that occurred also in *hPTX3/Galc^wt^* mice, possibly due to the PTX3 upregulation present in both groups (Figure S5). In keeping with these observations, the levels of transcription factor *Cebpd* were significantly reduced in the spinal cord of *hPTX3/Galc^twi/twi^* mice when compared to *Galc^twi/twi^* animals (Figure 7), no differences being instead observed between the two groups in the cerebrum and cerebellum (Figure S5). Accordingly, the levels of murine *Ptx3* transcript were reduced only in the spinal cord of *hPTX3/Galc^twi/twi^* animals (Figure 7). Thus, at variance with what was observed in the cerebrum (see above), the cumulative levels of the human *plus* murine PTX3 transcripts were reduced in the spinal cord of *hPTX3/Galc^twi/twi^* animals relative to the levels of murine *Ptx3* mRNA in *Galc^twi/twi^* mice (see Figure S4B).

## 3. Discussion

In humans, genetic deficiency of the sphingolipid-metabolizing enzyme GALC leads to Krabbe disease, a neuroinflammatory degenerative disorder. Here, we demonstrated that the soluble pattern recognition receptor PTX3 was expressed by monocyte-derived cells in brain specimens from Krabbe patients, its immunoreactivity being related to the extent of the pathology and gliosis. In keeping with these observations, *twitcher* mice, an authentic model of Krabbe disease [22,23], were characterized by the progressive upregulation of PTX3 along the CNS caudal-rostral axis, with no changes in the peripheral organs. The upregulation of the *Ptx3* transcript levels in cerebrum, cerebellum, and spinal cord of *twitcher* mice resulted in an increase in PTX3 protein levels in the affected tissues and plasma.

*Ptx3* expression can be induced in the CNS following stimulation by LPS or pro-inflammatory cytokines [16]. Accordingly, *Ptx3* upregulation is observed in mice under various experimental neuroinflammatory conditions, including neurotrauma [35], ischemia [18], limbic seizure [17] and autoimmune encephalomyelitis [36]. In addition, serum levels of PTX3 are increased in patients affected by neurodegenerative disorders, including Parkinson’s disease [21], ischemic stroke [20], and multiple sclerosis [36].

Our data extend these observations to Krabbe patients and demonstrate for the first time that *Ptx3* upregulation occurs in the CNS of *twitcher* mice in parallel with an increased expression of neuroinflammation-related genes, including various cytokines/chemokines, the marker of gliosis *Gfap*, the microglial marker *Iba1*, and the M1 macrophage polarization/microglia markers *CD16/CD32*. These changes were detectable at postnatal day P15, before the onset of evident neurologic signs, and were increased at P24, when pathologic alterations were occasionally noticeable, and at P35, when clear neurologic defects occurred, including tremor and hind limb paralysis [23,25,26]. These findings indicate that the inflammatory environment progressively established after birth in the CNS of GALC-deficient mice drives the expression of *Ptx3*, as confirmed by the observed upregulation of *Cebpd*, a transcription factor known to mediate *PTX3* expression in astrocytes during neuroinflammation [27]. 

Previous findings had shown that PTX3 can be expressed by neurons, astrocytes, and/or microglia following cytokine stimulation or under neuroinflammatory conditions, depending on the specific disorder and acute versus chronic phase of the disease (see [15,35,36] and references therein). Here, immunohistochemical analysis demonstrated that autopsy brain specimens from Krabbe infants were characterized by an intense gliosis and PTX3 immunoreactivity that was detectable in macrophages and globoid cells, a hallmark of Krabbe disease [9]. These observations were confirmed by the analysis of *Galc^twi/twi^* mice, in which PTX3 immunoreactivity was prevalently detectable in IBA1-positive globoid cells of the white matter of the brain cortex, cerebellum, and spinal cord of these animals, as well as in GFAP-positive astrocytes of the gray matter.

PTX3 is an important mediator of innate immune responses, produced locally at the site of inflammation [37]. In CNS, PTX3 modulates the activity of microglia by inhibiting phagocytosis of apoptotic cells and favoring the uptake of pathogens [24]. PTX3 binding helps the rescue of neurons from phagocytic clearance by macrophages [27] and protects them from ischemic damage [19,38], trauma [39], and in Parkinson’s disease [40]. Accordingly, neurogenesis and angiogenesis were inhibited after cerebral ischemia in *Ptx3* null mice, and neuronal damage was increased after limbic seizure when *Ptx3* deficient animals were compared to controls [17,18]. Together, these data point to a protective role of PTX3 during neuroinflammation.

Our data demonstrate that the constitutive overexpression of *hPTX3* that occurs from birth throughout the whole life of *hPTX3/Galc^twi/twi^* mice attenuates the severity of clinical signs, such as twitching and hind limbs clenching, and causes a significant, albeit limited, increase in their life span. However, when assessed at the late P35 stage of the disease, *hPTX3* overexpression did not exert any significant effect on the CNS of GALC-deficient mice in terms of IBA1-positive microglia infiltrate, GFAP-positive gliosis, and myelin degradation of MBP-positive fibers. In addition, a similar accumulation of the neurotoxic GALC substrate psychosine was observed in the cerebrum and spinal cord of *Galc^twi/twi^* and *hPTX3/Galc^twi/twi^* mice at this time point. These data appear to be in keeping with previous observations indicating that PTX3 is unable to exert a significant impact on the histopathological damage that occurs in mice after experimental neurotrauma or to affect the course of experimental autoimmune encephalomyelitis [35,36]. Nevertheless, we found that *hPTX3* overexpression reduced the upregulation of proinflammatory genes in the spinal cord of P35 *hPTX3/Galc^twi/twi^* mice when compared to *Galc^twi/twi^* animals, including endogenous *Ptx3* and its transcription factor *Cebpd*, with no effect on the neuroinflammatory response observed in the cerebrum and cerebellum of these animals. Relevant to this point, neurohistopathological and neurochemical alterations caused by GALC deficiency in the CNS of *twitcher* mice arise in a temporal and region-dependent fashion, the first alterations being observed in the spinal cord to progress along the caudal-rostral axis in the cerebellum and cerebrum (see [41] and references therein). In addition, spinal cord alterations were already observed in 18–21-week-old GLD fetuses [42,43]. Thus, the spinal cord represents the earliest tissue affected by GALC deficiency in murine and human CNS and appears to be more prone to a protective effect exerted by PTX3 on the neuroinflammatory response that occurs in *twitcher* mice. 

Different hypotheses can be raised to attempt to explain why the spinal cord is more responsive to the partial protecting role exerted by genetic, constitutive *hPTX3* upregulation in GALC-deficient mice. The ubiquitous production of PTX3 in *hPTX3* animals is under the control of the *Tie2* promoter [34] that drives the vascular expression of the transgene throughout embryogenesis and adulthood [44]. Thus, the early and long-lasting *PTX3* overexpression that occurs in *hPTX3/Galc^twi/twi^* animals may prevent, at least in part, the first lesions that arise in the spinal cord of GALC-deficient mice, being instead less effective against the later CNS lesions (further experiments performed on *hPTX3/Galc^twi/twi^* mice at earlier stages of the disease will be required to elucidate this point). In addition, the metabolic alterations that occur in parallel with the progression of gliosis, neurodegeneration, microglial activation, and apoptosis along the rostral-caudal axis in a regional and age-dependent fashion [41] may exert a different impact on the protective activity of PTX3 in *hPTX3/Galc^twi/twi^* mice. A further hypothesis can be based on the fact that PTX3 may also exert detrimental effects following CNS damage (see [15] and references therein), indicating that the activity of PTX3 in neuroinflammation may represent the result of a fine tuning in different areas of the CNS between pro- and anti-neurodegenerative mechanisms of action that remain poorly defined. Finally, other members of the long pentraxin family, represented by the neuronal pentraxins NP1, NP2 and NPR, may exert an impact on neurodegeneration and interfere with PTX3 activity [35]. Experiments performed in a *Ptx3* null background would be useful to assess these hypotheses. However, due to the female infertility of *Ptx3* null mice [45] and the early lethality of *Galc^twi/twi^* animals, the generation of double *Ptx3^−/−^/Galc^twi/twi^* mice would require a breeding program with a very large number of animals, incompatible with the rules of the local and national ethical committees. 

The neurotoxic GALC substrate psychosine has been used as a biomarker to identify patients with infantile GLD, and possibly late onset GLD patients, and to monitor the timing and efficacy of hematopoietic stem cell transplantation in these patients [46]. Our data demonstrate that serum levels of PTX3 increase in *twitcher* mice. Further studies will be required to assess whether longitudinal blood measurement of PTX3 levels may represent a novel biomarker more sensitive than psychosine to predict onset after newborn screening and to follow disease progression in transplanted and/or late-onset GLD patients.

In conclusion, our results provide the first evidence that PTX3 is produced in the CNS of Krabbe patients and GALC-deficient *twitcher* mice and may exert a partial protective role by reducing the neuroinflammatory response that occurs in the spinal cord of *Galc^twi/twi^* animals, with possible implications for GLD management and therapy.

## 4. Materials and Methods

### 4.1. Histopathology of Human GLD Biopsies

Autopsy brain specimens of GLD patients (Table S1) were obtained from the Program for Neurodevelopmental Function in Rare Disorders Brain and Tissue Bank, University of Pittsburgh School of Medicine. Use of this material was approved by the Committee for Oversight of Research and Clinical Training Involving Decedents (CORID) protocol No 583. Two matched control brains from 4-year-old and 11-year-old patients who died from unrelated non-neurological complications were obtained from the Archive of Pathological Department of Spedali Civili of Brescia. Their use was approved by the Ethics Board of Spedali Civili di Brescia (patient consent was not needed for retrospective and exclusively observational study on archival material obtained for diagnostic purposes (Delibera del Garante n. 52 del 24/7/2008 and DL 193/2003). 

Formalin-fixed, paraffin-embedded tissue sections were submitted to H&E and single or double immunohistochemical staining. Briefly, sections were de-waxed, rehydrated, and endogenous peroxidase activity blocked with 0.3% H_2_O_2_ in methanol for 20 min. Antigen retrieval was performed using a microwave oven or thermostatic bath in 1.0 mM EDTA buffer (pH 8.0). Sections were then washed in TBS (pH 7.4) and incubated for 1 h with the specific primary antibody diluted in TBS 1% bovine serum albumin. Signal was revealed using the DAKO Envision+System-HRP Labelled Polymer Anti-Mouse or Anti-Rabbit (Dako Cytomation, Santa Clara, CA, USA), followed by Diaminobenzydine (DAB) as chromogen and hematoxylin as counterstain. 

For double immunostains, after completing the first immune reaction, the second primary antibody was applied and labelled using MACH 4^TM^ Universal AP Polymer Kit (Biocare Medical, Pacheco, CA, USA); chromogen reaction was developed with Ferangi Blue^TM^ Chromogen System (Biocare Medical), and nuclei were faintly counterstained with Methyl Green. Images were then acquired with an Olympus DP70 camera mounted on an Olympus Bx60 microscope using AnalySIS imaging software (Soft Imaging System GmbH, Münster, Germany). The following primary antibodies were used: rabbit anti-PTX3 polyclonal antibody (1:500, kindly provided by B. Bottazzi Humanitas Clinical Institute, Rozzano, Italy), monoclonal mouse anti human CD68, clone PG-M1 (1:200, Dako Cytomation), and monoclonal mouse anti human GFAP, clone 6F2 (1:100, Dako Cytomation).

### 4.2. Animals

Breeder *twitcher* heterozygous mice (C57BL/6J, *Galc^twi/+^*; Jackson Laboratories, Bar Harbor, ME, USA) were maintained under standard housing conditions. Experiments were performed according to the Italian laws (D.L. 116/92 and following additions) that enforce the EU 86/109 Directive and were approved by the local animal ethics committee (OPBA, Università degli Studi di Brescia, Italy). *Twitcher* mutation was determined by polymerase chain reaction (PCR) on DNA extracted from clipped tails [47]. In all of the experiments, littermate wild type (*Galc^wt^*) and homozygous (*Galc^twi/twi^*) animals were used. To generate *PTX3*-overexpressing *twitcher* mice, syngeneic TgN(Tie2-hPTX3) male mice (*hPTX3* mice) that ubiquitously express human PTX3 under the control of the *Tie2* promoter [34] were bred with female *Galc^twi/+^* mice to obtain *hPTX3/Galc^twi/+^* animals. Next, *hPTX3/Galc^wt^* and *hPTX3/Galc^twi/twi^* mice were generated by crossing *hPTX3/Galc^twi/+^* breeder mice that were genotyped for *Galc* status by PCR and for *hPTX3* overexpression by RT-PCR. For the survival studies, the end point was established for each mouse by the animal house veterinarian unaware of the animal genotype, according to the rules of the local ethics committee.

### 4.3. Quantitative RT-PCR Analysis

Cerebrum, cerebellum, and spinal cord specimens were analyzed for gene expression by quantitative RT-PCR (qPCR) at P15, P24 and/or P35, and data were normalized for *Gapdh* expression [48]. For this purpose, total RNA was extracted from frozen samples using TRIzol Reagent according to the manufacturer’s instructions (Invitrogen, Carlsbad, CA, USA), and contaminating DNA was digested using DNAse (Promega, Madison, WI, USA). Total RNA (2 μg) was retrotranscribed with MMLV reverse transcriptase (Invitrogen) using random hexaprimers in a final 20 μL volume. Quantitative PCR was performed with a ViiA^TM^ 7 Real-Time PCR Detection System (Applied Biosystems, Waltham, MA, USA) using an iQTM SYBR Green Supermix (Biorad, Hercules, CA, USA) according to the manufacturer’s instructions. *Ptx3* expression levels were analyzed by qPCR also in kidneys, liver, and lungs from *Galc^wt^* and *Galc^twi/twi^* mice at P35. In each experiment, an arbitrary value equal to 1.0 was assigned to the levels of expression of the gene(s) measured in one sample that was used as reference. The specific primers are shown in Table S2.

### 4.4. Immunohistochemical Analysis

Formalin-fixed, paraffin-embedded tissue sections (7 µm) of the brain cortex, cerebellum, and spinal cord from P35 *Galc^wt^*, *Galc^twi/twi^*, *hPTX3*/*Galc^wt^* and *hPTX3*/*Galc^twi/twi^* mice were submitted to single or double immunohistochemical staining. For single staining, sections were incubated overnight at 4°C with the anti-PTX3 polyclonal antibody, the anti-Iba1 mouse monoclonal antibody (1:100; Genetex, Irvine, CA, USA), the anti-GFAP mouse monoclonal antibody, clone 6F2 (1:100; Dako), or the anti-Myelin Basic Protein MAB386 antibody (1:50, Millipore). Next, sections were incubated for 1 h with anti-Rabbit or anti-Mouse Envision+System-HRP Labelled Polymer (Dako) or with Rat-on Mouse HRP-Polymer (Biocare Medical) for myelin staining, followed by Diaminobenzydine (DAB) as chromogen and hematoxylin as counterstain.

For double immunostains, incubation with the anti-PTX3 polyclonal antibody was followed by 1 h incubation with biotin anti-rabbit antibody and by 1 h incubation with streptavidin Alexa Fluor 594. Sections were then incubated for 2 h with anti-GFAP or anti-Iba1 monoclonal antibody, followed by 1 h incubation with Alexa Fluor 488 anti-mouse antibody. All tissue sections were incubated for 30 min with DAPI for nuclear staining and mounted in Dako fluorescent mounting medium.

Images were taken with an Axiovert 200 M microscope equipped with ApoTome optical sectioning device (Carl Zeiss, Oberkochen, Germany) using the same settings for comparison of all samples. 

### 4.5. Western Blotting

Cerebrum, cerebellum, and spinal cord specimens from *Galc^wt^* and *Galc^twi/twi^* mice at P35 were used for Western blotting analysis. The tissues were homogenized in lysis buffer (1.0% NP-40, 20 mM Tris-HCl pH 8.0, 137 mM NaCl, 10% glycerol, 2.0 mM EDTA, 1.0 mM sodium orthovanadate, 10 mg/mL aprotinin, 10 mg/mL leupeptin). For PTX3 protein level analysis, tissue extracts (40 μg of protein) were probed with an anti-PTX3 polyclonal antibody. Monoclonal anti-vinculin antibody (Sigma-Aldrich, St Louis, MO, USA) was used as the loading control.

### 4.6. Serum PTX3

The blood levels of PTX3 were evaluated by ELISA (R&D Systems, Minneapolis, MN, USA) using 7.0 μL serum from *Galc^wt^* and *Galc^twi/twi^* mice at P28–34.

### 4.7. Assessment of Animal Clinical Features

Body weight, life span, twitching and hind limbs clenching were monitored daily from day P8 until mice reached a moribund condition. Each mouse was observed for at least 1 min by two trained observers. The extent of frequency and severity of twitching was scored as: 1, fine; 2, mild; 3, mild-moderate; 4, moderate; 5, severe. Hind limbs clenching frequency was scored: 1, rare; 2, mild; 3, intermittent; 4, moderate; 5, severe [49].

### 4.8. Psychosine Extraction and MS Analysis

Psychosine extraction was performed as previously described [50]. Briefly, 20 mg of lyophilized brain or spinal cord were extracted with 15 mL of acetone using a Polytron homogenizer to selectively extract psychosine. The extract was filtered through a Büchner funnel and evaporated to dryness. The dried residue was dissolved in 2.0 mL chloroform/methanol (2/1 *v*/*v*), and the psychosine-enriched fraction was evaporated to dryness, dissolved in methanol, and further analyzed by mass spectrometry (MS). 

All MS analyses were performed at the Unitech OMICs platform (University of Milano, Italy) using an ExionLC™ AD system connected to TripleTOF™ 6600 System equipped with Turbo V™ Ion Source with ESI Probe (SCIEX, Framingham, MA, USA). Samples were separated on a Kinetex^®^ EVO C18 100 (Length) × 2.1 mm (ID) × 1.7 µm (particle size). The temperature was set at 40 °C. The analytes were eluted with the following gradient: from 60% buffer A (0.01% formic acid in water) to 99% buffer B (0.01% formic acid in methanol) in 7.5 min. Constant flow rate: 450 µL/min. Total run: 12 min. MS spectra were collected, in positive polarity, in full-mass scan from 250 to 800 Da (100 ms accumulation time) and in IDA^®^ mode (information-dependent acquisition) from 100 to 800 Da (40 ms accumulation time, top 18 spectra per cycle 0.87 s). Nitrogen was used as a nebulizing gas (GS1, 55 psi), turbo spray gas (GS2, 65 psi), and curtain gas (CUR, 35 psi). Spray voltage was fixed at 5.0 kV, de-clustering potential (DP) was 50 eV, the collision energy was 30 eV with a collision energy spread (CES) of 15 eV, and source temperature was 300 °C.

The data were analyzed using SCIEX OS 1.4 software (SCIEX™), together with LibraryView™ (version 1.0) containing the hexosylsphingosine mass spectra. 

### 4.9. Statistical Analysis

Comparisons among multiple groups were performed using a one-way ANOVA model, followed by post hoc comparisons with adjustment for multiple comparisons (Sidak procedure). Comparison between two groups was performed using the Student’s unpaired *t*-test.

## Figures and Tables

**Table 1:** GFAP/PTX3 immunostaining in Krabbe patients.

Deidentified Case ID	* Pathology	^§^ GFAP+	^§^ PTX3+
CW16 064	3 (severe)	2 (gliosis)	2 (mainly in perivascular globoid cells)
CW17 060	3 (severe)	2 (gliosis)	2 (mainly in perivascular globoid cells)
CW18 060	3 (severe)	2 (gliosis)	2 (mainly in perivascular globoid cells)
CW16 066	3 (severe)	2 (gliosis)	2 (mainly in perivascular globoid cells)
CW16 061	2 (moderate)	2 (gliosis)	2 (mainly in perivascular globoid cells)
CW16 065	1 (mild to moderate)	2 (gliosis)	1 (mainly in perivascular globoid cells)
CW18 064	1 (modest)	2 (gliosis)	1 (only few inflammatory cells)
CW16 062	0 (no evidence)	1 (moderate gliosis)	1 (only few inflammatory cells)
CW15 103	0 (no evidence)	1 (mild gliosis)	0 (no inflammatory cells)

* Perivascular globoid cells: 0, no cells; 1, rare cells; 2, moderate number of cells; 3, high number of cells. **^§^** GFAP and PTX3 semiquantitative immunoreactivity score: 0, no immunoreactivity; 1, mild immunoreactivity; 2, moderate immunoreactivity.

**Table 2:** Psychosine levels in the CNS of GALC-deficient mice.

	Cerebrum *	Spinal Cord *
*hPTX3* mice.	5.7 ± 2.8	1.5 ± 0.8
*Galc^twi/twi^* mice	514.4 ± 288.1	281.6 ± 38.1
*hPTX3/Galc^twi/twi^* mice	552.0 ± 90.9	393.2 ± 170.1

* Psychosine levels were assessed at P35 and normalized to the levels measured in *Galc^wt^* mice. Data are the mean ± S.E.M. of 3 animals per group.

## Data Availability

Not applicable.

## Supplementary Materials

The following supporting information can be downloaded at: https://www.mdpi.com/article/10.3390/ijms23169436/s1.
