# Supplementary material for: β-Galactosylceramidase Deficiency Causes Upregulation of Long Pentraxin-3 in the Central Nervous System of Krabbe Patients and Twitcher Mice
Source: Int J Mol Sci. 2022 Aug 21;23(16):9436. doi: 10.3390/ijms23169436 (PMC9409448; doi:10.3390/ijms23169436)
Supplement: Supplementary file 1 [file ijms-23-09436-s001.zip › ijms-1861502-supplementary.pdf]

Supplementary Materials

Table S1. Krabbe patients

Deidentified case ID	Age of Onset	Asymptomatic	Krabbe disease Classification	Transplanted	Age of transplant	Initial symptoms after transplant if asymptomatic	Age of death	Stage
CW15-103	N/A	Yes	Early Infantile	Yes	1 month 6 days	6 months 17 days	15 years 5 months 30 days	N/A
CW16-061	5 months	No	Early Infantile	No	N/A	N/A	6 years 9 months 12 days	IV at 19 months
CW16-064	3.5 months	No	Early Infantile	No	N/A	N/A	1 year 6 months	II-III at 5 months
CW17-060	4 months	No	Early Infantile	No	N/A	N/A	1 year 2 months 13 days	IV at 10 months
CW18-060	5 months	No	Early Infantile	No	N/A	N/A	3 years 1 month 8 days	ND
CW18-064	8 months	No	Late Infantile	No	N/A	N/A	12 years 5 months	II at 3 years 10 months
CW16-062	12 months	No	Late Infantile	No	N/A	N/A	9 years 8 months 20 days	IV at 5 years 7 months
CW16-065	8 months	No	Late Infantile	No	N/A	N/A	2 years 4 months 18 days	IV at 20 months
CW16-066	9 months	No	Late Infantile	No	N/A	N/A	2 years 2 months 9 days	IV at 22 months

N/A= Not applicable, ND= No Data
Stage II- increased tone irritability, difficulty eating; Stage III- hypotonia, unable to eat; Stage IV- blind, deafness and quadriplegia, dysautonomia.

Table S2. qPCR oligonucleotide primers

Gene	Forward	Reverse
Ptx3	5'-GACCTCGGATGACTACGAG	5'-CTCCGAGTGCTCCTGGCG
Ptx3+hPtx3	5'-TTTGTGCTCTCTGGTCTGC	5'-CGAGTTCTCCAGCATGATGAA
Mbp	5'-GCTTCTTTAGCGGTGACAGG	5'-CTTGGGATGGAGGTGGTGT
Cepbd	5'-TCGACTTCAGCGCCTACATT	5'-CTAGCGACAGACCCCACAC
Tnfa	5'-GCCTCTTCTCATTCCTGCTT	5'-TGATCTGAGTGTGAGGGTCTG
Cxcl1	5'-GCTGGGATTCACCTCAAGAA	5'-TGGGGACACCTTTTAGCATC
IL1a	5'-CAGTTCTGCCATTGACCATC	5'-GAATCTTCCCGTTGCTTGAC
Gfap	5'-AGAAAACCGCATCACCATTC	5'-CGTCCTTGTGCTCCTGCT
Iba1	5'-CGAATGCTGGAGAAACTTGG	5'-ACCAGTTGGCCTCTTGTGTT
CD16/32	5'-CTGGGAGTGATTTCTGACTGG	5'-TGGTTGGCTTTTGGGATAGA
Arg1	5'-GCAGAGGTCCAGAAGAATGG	5'-TTGTCAGGGGAGTGTTGATG
CD206	5'-TGGCGAGCATCAAGAGTAAA	5'-CATAGGAAACGGGAGAACCA
Gapdh	5'-GAAGGTCGGTGTGAACGGATT	5'-TGACTGTGCCGTTGAATTTG

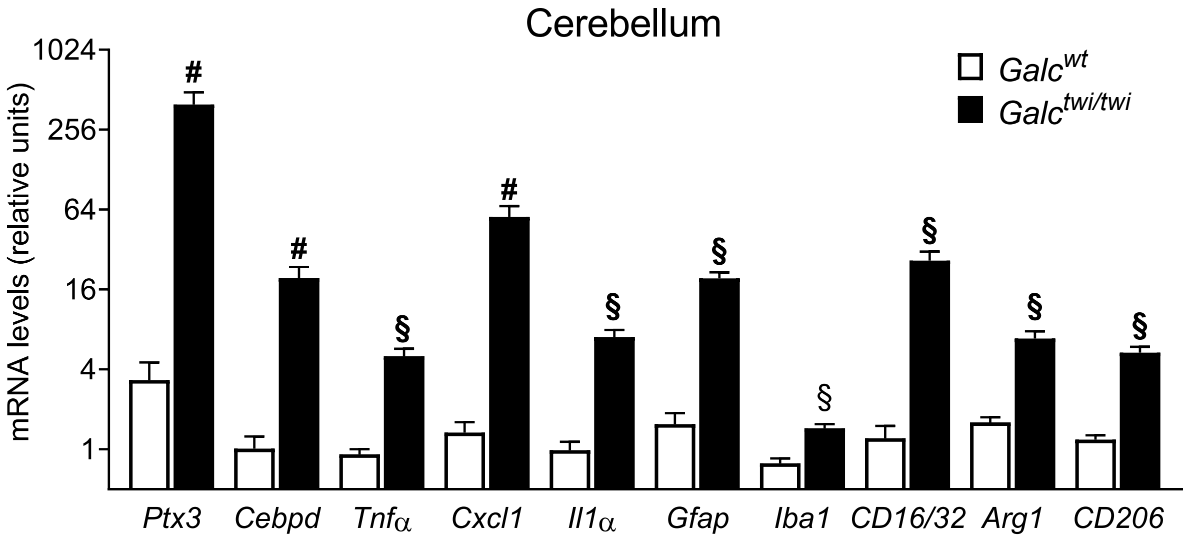

Figure S1. *Ptx3* and proinflammatory gene expression in the cerebellum of *twitcher* mice. Steady state mRNA levels of the indicated genes were evaluated by qPCR in the cerebellum of $Galc^{wt}$ and $Galc^{twi/twi}$ mice harvested at P35. Data were normalized to *Gapdh* expression and are the mean ± SEM of 7-10 animals per group, #, $P < 0.01$; §, $P < 0.001$, $Galc^{wt}$ *versus* $Galc^{twi/twi}$, Student's t-test.

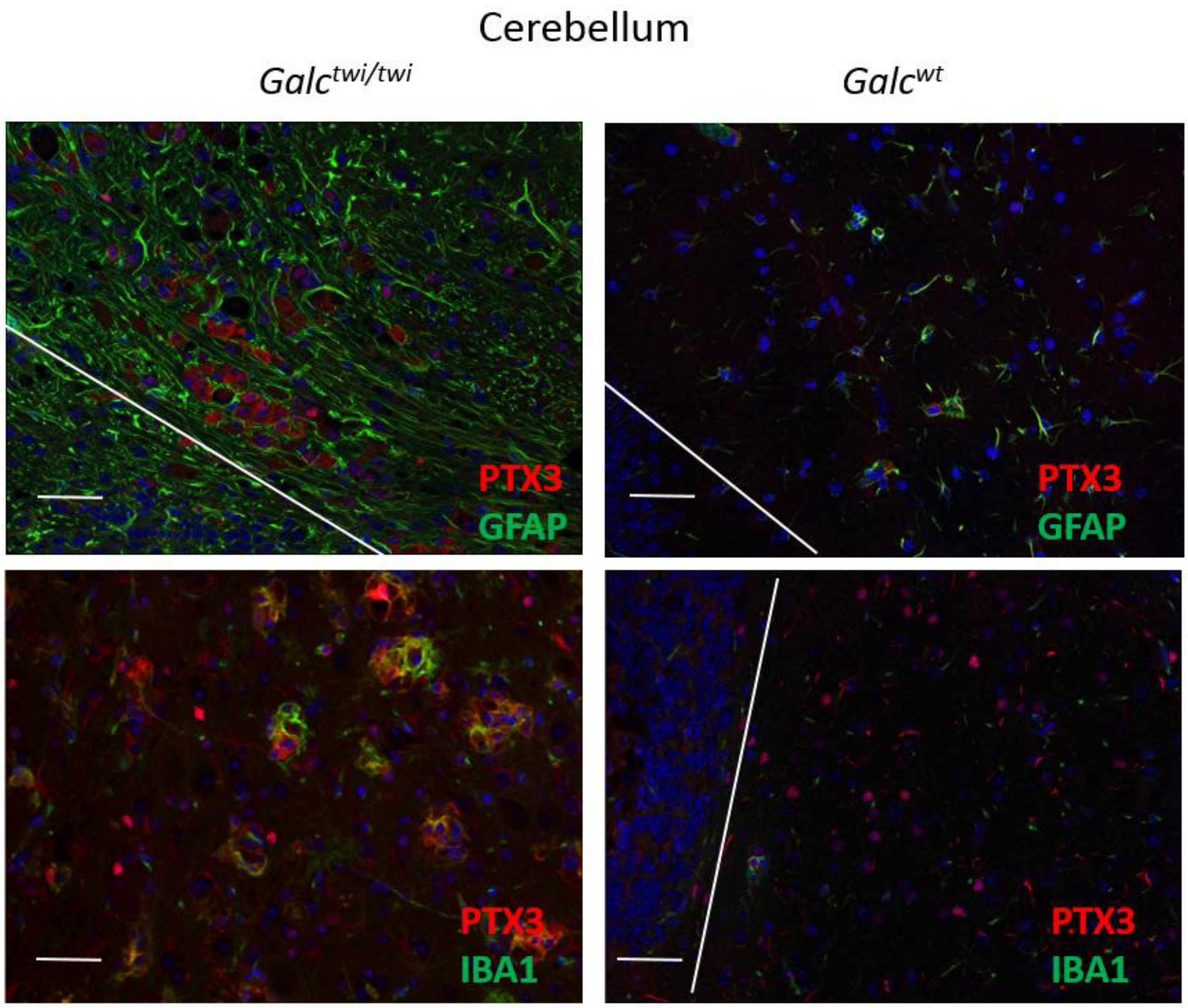

Figure S2. PTX3 immunolocalization in the cerebellum of *Galc*$^{twi/twi}$ *and Galc*^{wt} mice. Paraffin-embedded sections of cerebellum of *Galc*$^{twi/twi}$ and *Galc*^{wt} mice were double-immunostained at P35 with anti-PTX3/GFAP or anti-PTX3/IBA1 antibodies. The white line indicates the boundary between white and gray matter. Scale bar, 50 μm.

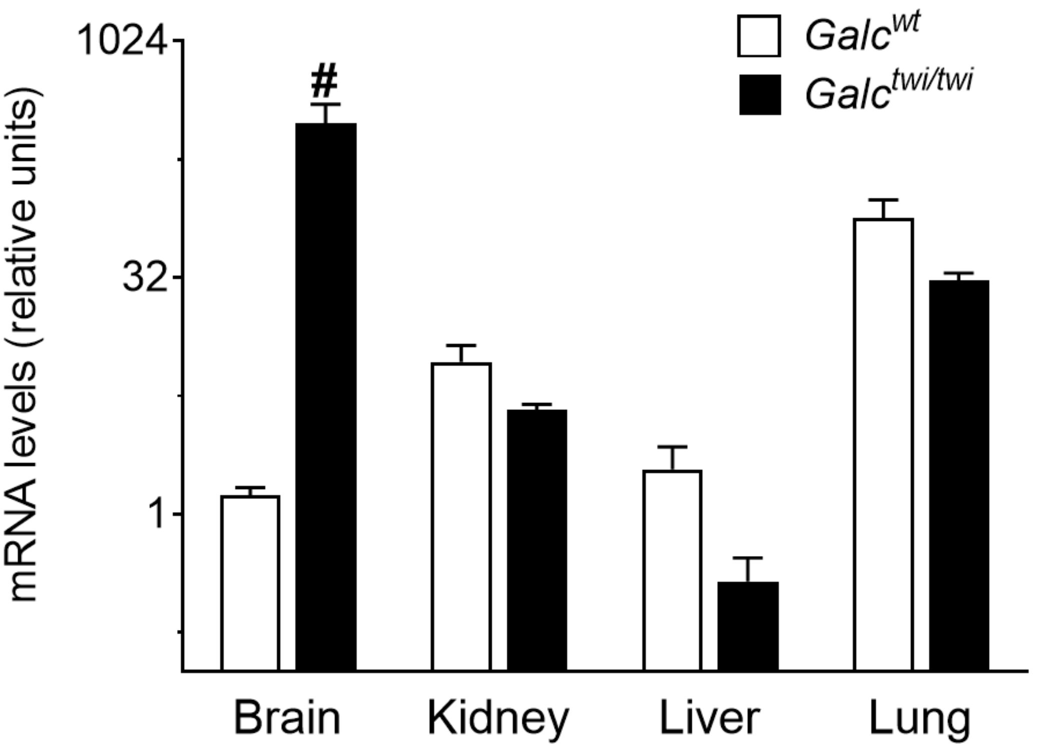

Figure S3. ***Ptx3*** **expression in the peripheral organs of** ***twitcher*** **mice.** *Ptx3* expression was evaluated by qPCR in the brain, kidney, liver, and lungs of $Galc^{wt}$ and $Galc^{twi/twi}$ mice at P35. Data are the mean ± S.E.M. of 3-6 animals per group. #, P < 0.01, $Galc^{wt}$ *versus* $Galc^{twi/twi}$, Student's t-test.

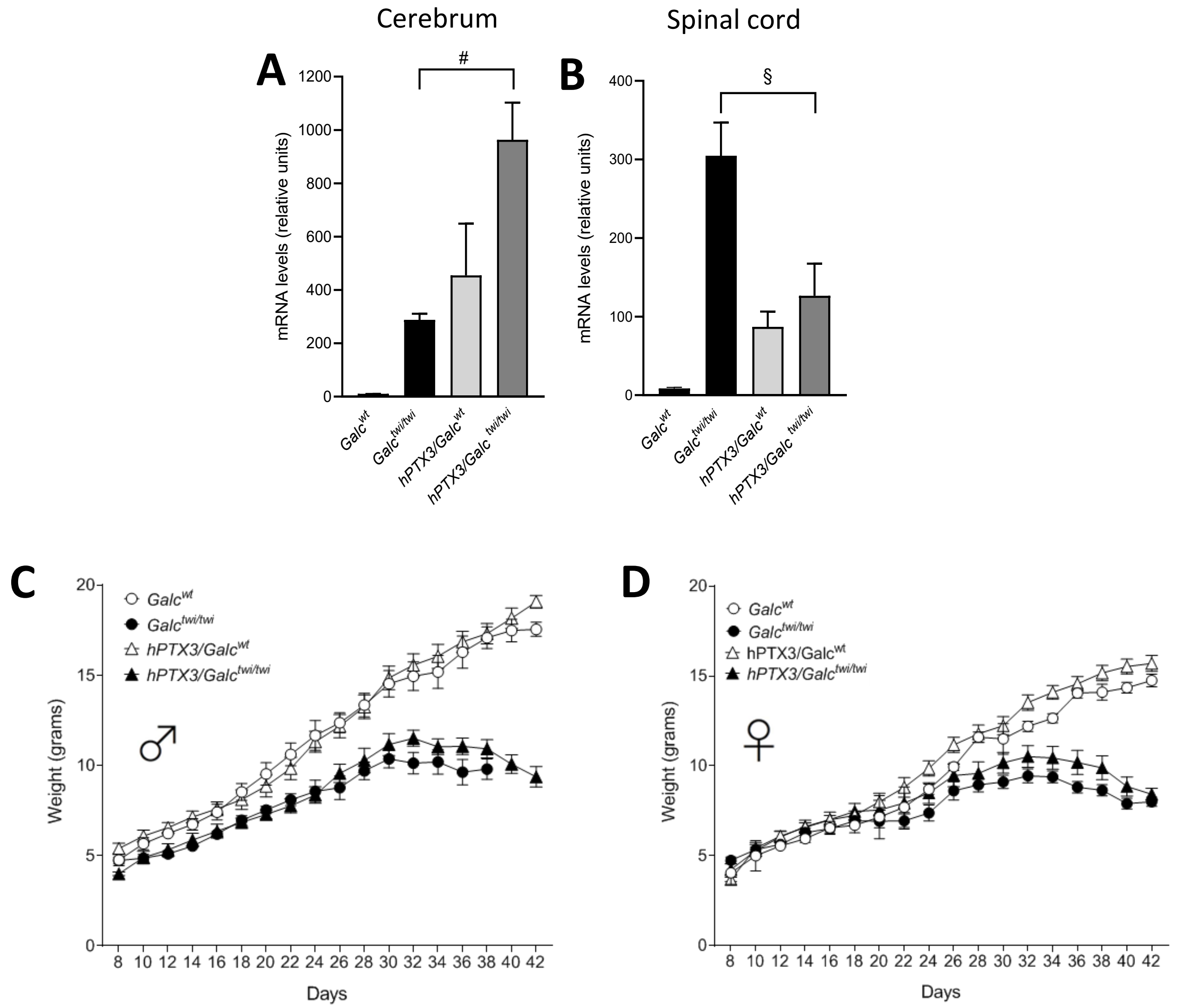

Figure S4. Characterization of *hPTX3/Galc*$^{twi/twi}$ mice. A, B) qPCR analysis of murine *plus* human PTX3 expression in cerebrum (**A**) and spinal cord (**B**) of *hPTX3/Galc*$^{twi/twi}$ mice compared to *Galc*^{wt}, *Galc*$^{twi/twi}$ and *hPTX3/Galc*^{wt} animals. The analysis was performed at P35 using oligonucleotide primers designed to recognize simultaneously both human and murine PTX3 transcripts. Data are the mean ± SEM. of 3-4 animals per group. #, $P < 0.01$; §, $P < 0.05$, Student's t-test. **C, D**) The body weight gain of male (**C**) and female (**D**) hPTX3 overexpressing *hPTX3/Galc*^{wt} and *hPTX3/Galc*$^{twi/twi}$ mice was compared to that of the corresponding control *Galc*^{wt} and *Galc*$^{twi/twi}$ animals. Data are the mean ± SEM. of 2-12 animals per group.

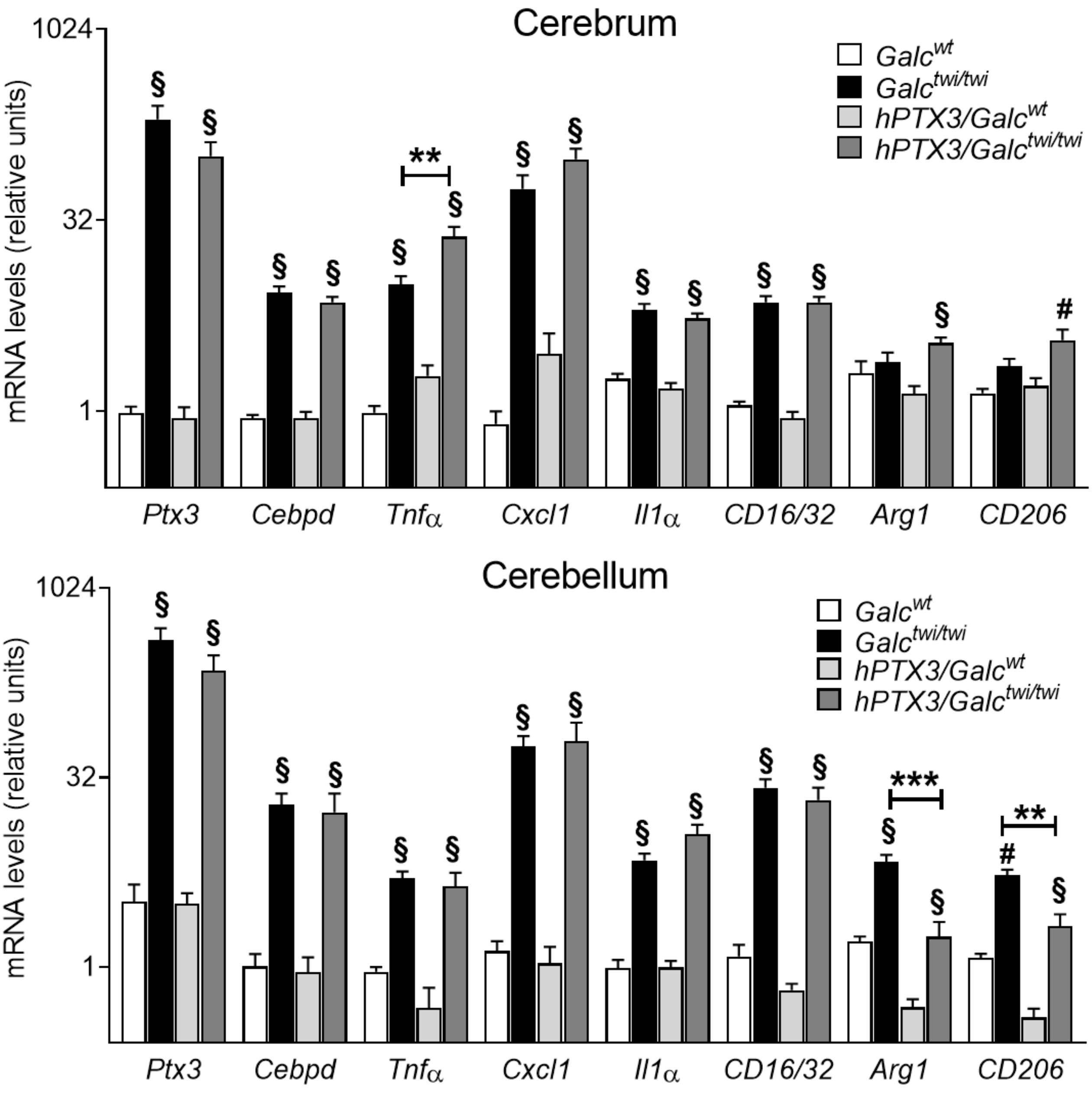

Figure S5. qPCR analysis of the cerebrum and cerebellum of *hPTX3/Galc*^{wt} and *hPTX3/Galc*$^{twi/twi}$ mice. Steady state mRNA levels of the indicated genes were evaluated by qPCR in the cerebrum and cerebellum of *hPTX3/Galc*^{wt} and *hPTX3/Galc*$^{twi/twi}$ mice and compared to those measured in the corresponding control *Galc*^{wt} and *Galc*$^{twi/twi}$ mice harvested at P35. Data were normalized to *Gapdh* expression and are the mean ± SEM. of 7-10 animals per group. **, P < 0.01; ***, P < 0.001, for *Galc*$^{twi/twi}$ *versus hPTX3/Galc*$^{twi/twi}$; #, P < 0.01; §, P < 0.001 for *Galc*^{wt} *versus Galc*$^{twi/twi}$ or hPTX3/*Galc*^{wt} *versus hPTX3/Galc*$^{twi/twi}$. One-way ANOVA with post-hoc comparisons with adjustment for multiple comparisons (Sidak).

Figure 3B

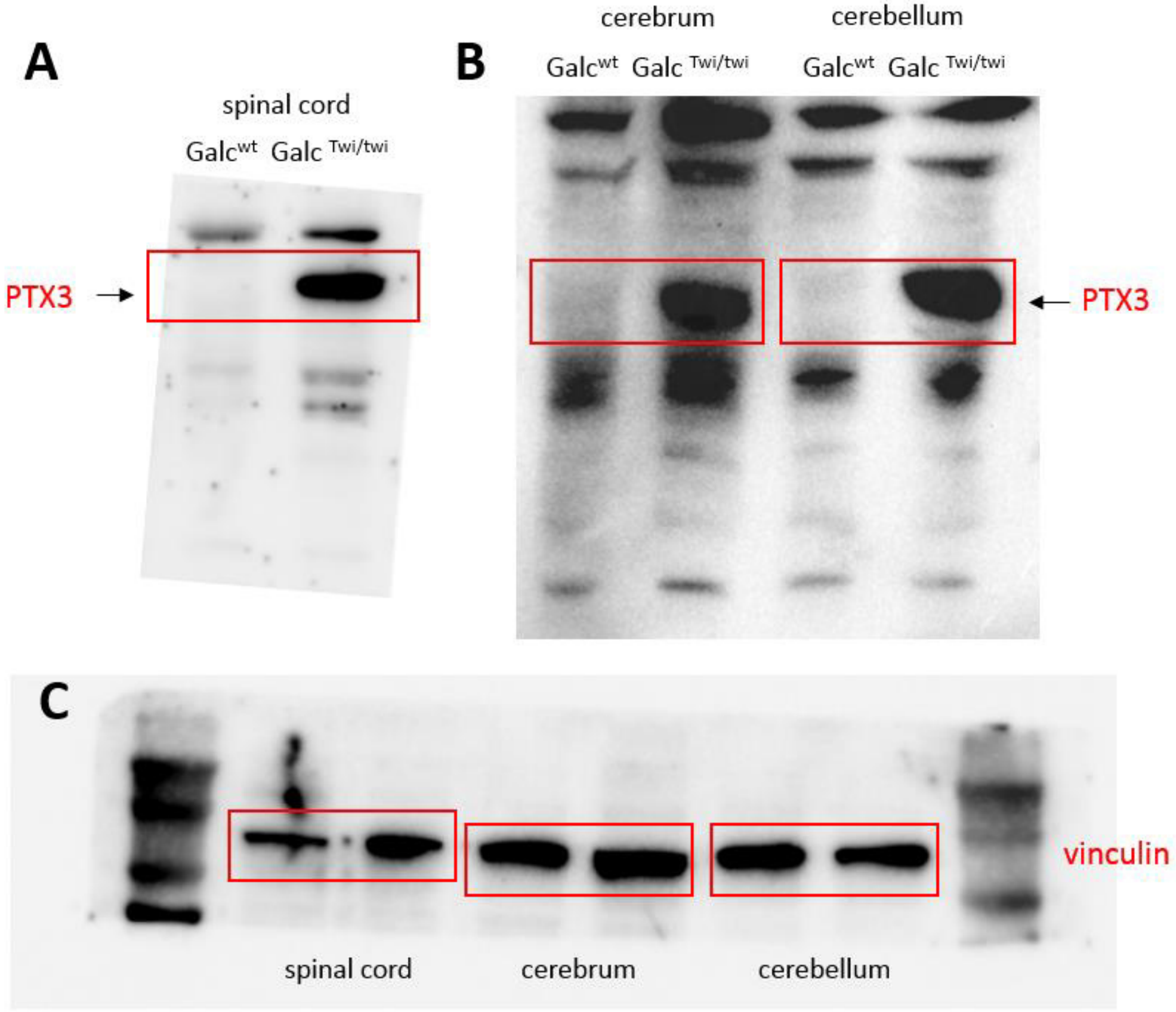

Addendum to Figure 3B. Spinal cord (**A**), cerebrum and cerebellum (**B**) extracts were run on separate gels and decorated with anti-PTX3 antibody. The same extracts were run also on a separate gel that was decorated with an anti-vinculin antibody (**C**).
